# Correction: Lyons-Weiler, J., et al. Relative Incidence of Office Visits and Cumulative Rates of Billed Diagnoses along the Axis of Vaccination. *Int. J. Environ. Res. Public Health* 2020, *17*, 8674

**DOI:** 10.3390/ijerph18030936

**Published:** 2021-01-22

**Authors:** James Lyons-Weiler, Paul Thomas

**Affiliations:** 1 The Institute for Pure and Applied Knowledge, Pittsburgh, PA 15101, USA; 2 Integrative Pediatrics, Portland, OR 97225, USA; paulthomasmd@drpaul.md

## Abstract Correction

There were two errors in the original article [1]. The ADHD rate reported in the Abstract in the vaccinated should be 5.3%, not 0.063% The error was due to counting office visits instead of incidence and failing to convert the probability to a percentage. Similarly, the correct study-wide ASD rate reported is 0.361%, not 0.84% The error was due to counting office visits instead of incidence, but the conversion of probability to percentage was correct. The US national rate of ADHD is 9.3% (CDCa), and the national rate of ASD is 1.851% (CDCb). Accordingly, the corrections have been made to the Abstract. We thank an astute reader for their input on this matter.

**Abstract:** We performed a retrospective analysis spanning ten years of pediatric practice focused on patients with variable vaccination born into a practice, presenting a unique opportunity to study the effects of variable vaccination on outcomes. The average total incidence of billed office visits per outcome related to the outcomes were compared across groups (Relative Incidence of Office Visit (RIOV)). RIOV is shown to be more powerful than odds ratio of diagnoses. Full cohort, cumulative incidence analyses, matched for days of care, and matched for family history analyses were conducted across quantiles of vaccine uptake. Increased office visits related to many diagnoses were robust to days-of-care-matched analyses, family history, gender block, age block, and false discovery risk. Many outcomes had high RIOV odds ratios after matching for days-of-care (e.g., anemia (6.334), asthma (3.496), allergic rhinitis (6.479), and sinusitis (3.529), all significant under the Z-test). Developmental disorders were determined to be difficult to study due to extremely low prevalence in the practice, potentially attributable to high rates of vaccine cessation upon adverse events and family history of autoimmunity. Remarkably, zero of the 561 unvaccinated patients in the study had attention deficit hyperactivity disorder (ADHD) compared to 5.3% of the (partially and fully) vaccinated. The implications of these results for the net public health effects of whole population vaccination and with respect for informed consent on human health are compelling. Our results give agency to calls for research conducted by individuals who are independent of any funding sources related to the vaccine industry. While the low rates of developmental disorders prevented sufficiently powered hypothesis testing, it is notable that the overall rate of autism spectrum disorder (0.361%) in the cohort is one-fifth that of the US national rate (1.851%). The practice-wide rate of ADHD was roughly half of the national rate. The data indicate that unvaccinated children in the practice are not unhealthier than the vaccinated and indeed the overall results may indicate that the unvaccinated pediatric patients in this practice are healthier overall than the vaccinated.

CDCa. Data and Statistics about ADHD. Available online: https://www.cdc.gov/ncbddd/adhd/data.html (accessed on 9 January 2021). 

CDCb. Data & Statistics on Autism Spectrum Disorder. Available online: https://www.cdc.gov/ncbddd/autism/data.html (accessed on 9 January 2021).

The authors apologize for any inconvenience caused and state that the scientific conclusions are unaffected. The original article has been updated.
